# An FPGA-Based Machine Learning Tool for In-Situ Food Quality Tracking Using Sensor Fusion

**DOI:** 10.3390/bios11100366

**Published:** 2021-09-30

**Authors:** Daniel Enériz, Nicolas Medrano, Belen Calvo

**Affiliations:** Faculty of Science, University of Zaragoza, 50009 Zaragoza, Spain; nmedrano@unizar.es (N.M.); becalvo@unizar.es (B.C.)

**Keywords:** e-nose, food quality, TVC, sensor fusion, neural networks, FPGA

## Abstract

The continuous development of more accurate and selective bio- and chemo-sensors has led to a growing use of sensor arrays in different fields, such as health monitoring, cell culture analysis, bio-signals processing, or food quality tracking. The analysis and information extraction from the amount of data provided by these sensor arrays is possible based on Machine Learning techniques applied to sensor fusion. However, most of these computing solutions are implemented on costly and bulky computers, limiting its use in in-situ scenarios outside complex laboratory facilities. This work presents the application of machine learning techniques in food quality assessment using a single Field Programmable Gate Array (FPGA) chip. The characteristics of low-cost, low power consumption as well as low-size allow the application of the proposed solution even in space constrained places, as in food manufacturing chains. As an example, the proposed system is tested on an e-nose developed for beef classification and microbial population prediction.

## 1. Introduction

**Figure 1:**
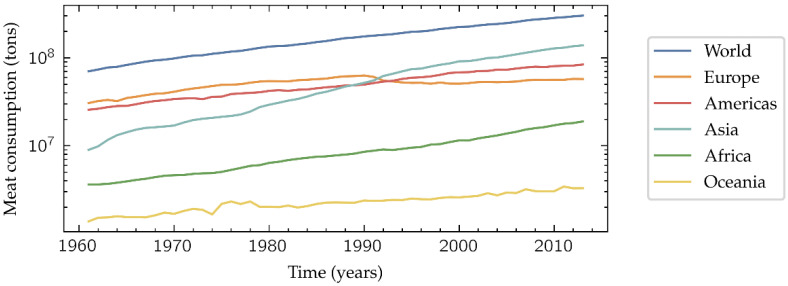
Meat consumption in tons per year. Data from FAOSTAT [2].

The world population growth results in an increasing global meat consumption (Figure 1). This tendency is especially marked in middle-income countries, where the quality and safety of meat products have been a public health concern due to the spoilage during distribution [1]. Additionally, the grade of acceptable spoilage is often subjectively judged, since it may be culturally and/or economically influenced. Therefore, it would be desirable the development of an objective, fast and low-cost method to determine if a meat sample is suitable for its intake or not, ensuring the basic levels of quality and safety when it reaches the final consumer.

One of the most popular indicators [3] of meat spoilage is the Total Viable Count (TVC), a quantitative estimation of the concentration of microorganisms in a sample, in this case of meat. Traditionally this indicator has been estimated using sophisticated methods as hyperspectral imaging [4,5], Fourier-Transform Infrared (FTIR) spectroscopy [6], or Polymerase Chain Reaction (PCR) [7]. Nevertheless, in recent years there have been approaches that use low-cost methods based on the detection of Volatile Organic Compounds (VOCs) emitted in the biochemical processes occurring during the meat spoilage [8]. A convenient solution is the VOC-reactive dyes, that change their color in presence of spoilage-related VOCs [9], enabling a naked-eye visualization of the meat quality. Another low-cost solution that has demonstrated to be successful in VOCs detection for TVC estimation are e-noses, i.e., an array of chemical gas sensors with a sensorial fusion processing system based on Machine Learning (ML) methods such as Principal Component Analysis (PCA) [10,11], Support Vector Machines (SVM) [10], fuzzy logic [12], Artificial Neural Networks (ANN) [11,12], k-Nearest Neighbors (k-NN) [13], or Information-Theoretic Ensemble Feature Selection (ITEFS) [14].

Another point that should be considered in the development of a meat quality determination method is the device that is going to process the data and interface to the user, since it is meant to be used along all the distribution chain: production centers, logistics platforms, and markets. Focusing on the e-noses, there are two options for running the aforementioned ML algorithms: cloud computing or edge computing. Although the first one requires the device to have less computational resources, it also needs to be actively connected to the Internet, with an extra battery consumption, limiting the places of operation, and with a risk of loss of data privacy. On the other hand, an edge device could be a better solution since it eliminates the issues related to the internet connection at the cost of a computationally stronger hardware.

There are various hardware options in which the ML algorithms can be run on the edge, ranging from the general-purpose Central Processing Units (CPUs), that allow a limited parallelism in their operation and are easy to program, to the Application-Specific Integrated Circuits (ASICs), that their custom design makes them to achieve the best performance. In a middle point, the Field-Programmable Gate Arrays (FPGAs) consist of a matrix of Configurable Logic Blocks (CLBs) which interconnections can be reprogrammed to meet desired application or functionality requirements after manufacturing. As FPGAs logic architecture can be customized meeting requirements of specific applications, providing good performance, these devices are becoming very popular for ML implementations in recent years [15,16].

**Figure 2:**
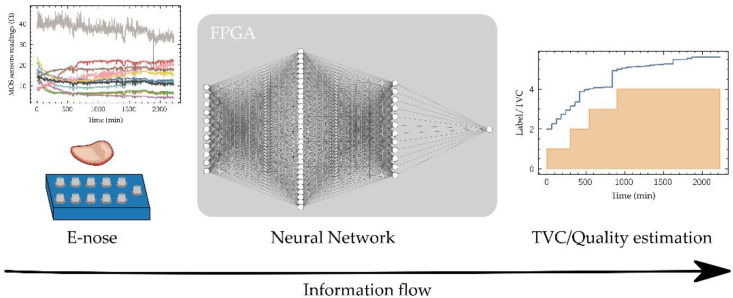
Developed system scheme. The readings of the e-nose feed the FPGA-implemented neural network that allow the estimation of the TVC and the quality label.

This project proposes a workflow to develop an edge-computing device for in-situ food quality tracking using sensor fusion powered by ANN, paying particular attention to the selection of the fixed-point numerical representation of data, network parameters and the corresponding logic/arithmetic blocks architecture thus keeping hardware requirements low enabling its implementation in a low-cost FPGA. To validate this workflow, an ANN model has been trained over a 12 beef cuts quality dataset [17] to estimate the TVC using the readings of an e-nose composed of 11 Metal-Oxide-Semiconductor (MOS) sensors (Figure 2). Once trained, the network has been implemented in a low-cost FPGA using fixed-point representation, which features easily allow its location in any place along the production-distribution chain. After the Introduction, the paper is organized as follows: Materials and Methods used in this work are detailed in Section 2, presenting the dataset and describing the ML-based architecture selected for data processing and its main characteristics in order to reduce its complexity, as well as the selection of the hardware where the proposed model will be implemented; Section 3 shows the Results achieved along the different stages of development of the model, from the high-level implementation as a computer software for model training, to its simulation in low-accuracy number representation, and model mapping into the hardware. Finally, Section 4 summarizes the main conclusions of this work.

## 2. Materials and Methods

With the objective of developing an edge-computing device for food quality tracking using sensor fusion, this section presents the details of the dataset used, as well as the network architecture characteristics. Then the training process is described, followed by the FPGA implementation, with the details of fixed-point selection method, model retraining, targeted FPGA, synthesis, and hardware implementation test.

### 2.1. Dataset

**Figure 3:**
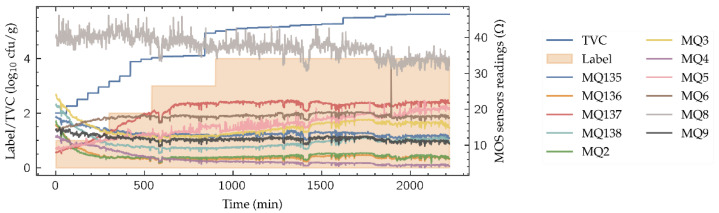
E-nose signals for the Inside/Outside cut with the TVC and the quality label.

Figure 3 shows part of the dataset used in this work, available in [17]. It is composed by 12 time-series’ of 2220 min long, corresponding to 12 beef cuts. Each time series contains the TVC evolution during the spoilage process, the quality label and the 11-sensors e-nose readings, all of them monitored every minute, thus having 2220 samples for each cut. The beef cuts are the following: Round (Shank), Top Sirloin, Tenderloin, Flap Meat (Flank), Striploin (Shortloin), Brisket, Clod/Chuck, Skirt Meat (Plate), Inside/Outside, Rib Eye, Shin and Fat. The real TVC is the target variable for the neural model. It was acquired using two different methods that were combined: the optical density estimation using a spectrometer and the microbial population measure with a hemocytometer. Note that the resolution of the TVC obtained (Figure 3) does not allow to detect changes every time step, thus presenting a stepped behavior. The discrete quality label ranges from 1 to 4, denoting “excellent” (label value 1), “good”, “acceptable” and “spoiled” (label value 4) depending on the TVC values. The e-nose consists on an 11 MOS sensor array, namely sensors MQ135, MQ136, MQ137, MQ138, MQ2, MQ3, MQ4, MQ5, MQ6, MQ8 and MQ9, whose sensitivities are available in Table 1. These gas sensors are chemoresistive, meaning that their detection principle is based on the change of sensor resistance when the sensible gases come in contact with the sensing material.

To reduce the high noise levels, present in the sensor readings (Figure 3) and its effect in the system performance, a Savitzky–Golay convolutional filter (window length 15, polynomial order 5) has been applied to the readings of the MOS sensors in the whole dataset. Savitzky-Golay convolutional filters have demonstrated its suitability in other e-nose ML-based processing systems [18,19]. After the data filtering, each of the e-nose sensor outputs and the TVC values have been normalized along all the dataset using the following expression:

(1) $$x^{\prime }=\frac{x-x_{\min}}{x_{\max}-x_{\min}}$$

where *x* is the variable to be normalized, $x_{\min}$ its minimum, $x_{\max}$ its maximum and *x*’ the normalized variable.

### 2.2. Neural Network Architecture

**Figure 4:**
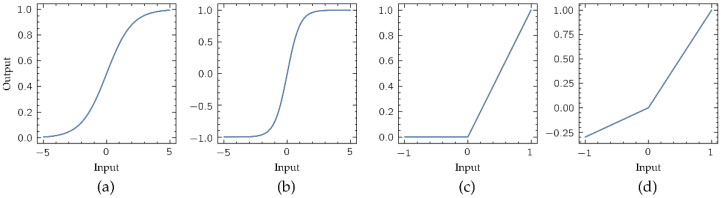
Most common non-linear activation functions: (**a**) sigmoid, (**b**) hyperbolic tangent, (**c**) Rectified Linear Unit (ReLU), and (**d**) Leaky ReLU (LReLU). Unlike the ReLU function, the LReLU presents a negative slope in the output for the negative values in its input. Note that the negative slope shown here is 0.3 for display purposes, although the used in this work is 0.01.

The process of estimating the TVC value from the sensors’ readings provided by an e-nose can be considered a regression modelling. Since the final target is to implement the developed model on a low-cost small-size processing device, the selected model architecture is a fully connected ANN, also known as Multi-Layer Perceptron (MLP). MLP architecture already widely has demonstrated its capability in e-nose data processing [20,21,22] at a relatively low computational cost. In this work, the selected MLP architecture consists of two hidden layers of 32 and 12 neurons with a total of 793 parameters (Figure 2). The processors (neurons) used in is type of ML algorithm are characterized by the following mathematical operation between layers:

(2) y=f(Wx+b)

where x is the input vector to a processors layer, W the weight matrix that modulates the effects of this input vector in the layer output, b the bias vector, and y the layer output vector. Function f(.) is the activation function, that provides the output of the layer processors. Activation function is usually defined as non-linear, making possible modelling more complex relations between inputs and outputs. Classical output functions in these ANN models are the sigmoid and hyperbolic tangent functions (Figure 4a,b, respectively). However, due the implementation of these functions requires high computational resources, its replacement by simpler non-linear functions is mandatory for its implementation in portable low-complexity computing devices. In this work the activation function selected is the Leaky Rectified Linear Unit (LReLU) (Figure 4d), a variant of the popular Rectified Linear Unit (ReLU) (Figure 4c) defined by the following expression [23]:

(3) $$\mathrm{LReLU}\left(x\right)=\{\begin{array}{c} \;x\;\;\;\;\;\mathrm{if}\;x>0\\ 0.01x\;\;\mathrm{if}\;x\leq 0 \end{array}\;$$

The choice of the LReLU instead of the ReLU prevents the ‘dying ReLU’ problem [24], that limits the convergence of a ML model during training.

### 2.3. Training and Validation Processes

In order to reduce the size of the ML model to be implemented in the selected processing device, thus facilitating that it fits in the available resources, different neural models will be developed for each of the 12 beef cuts available in the data set, keeping the same basic architecture (number of layers and processors per layer), and fitting different weight set values to each of the corresponding sub-datasets. This approach assumes that the end-user will know the beef cut monitored in every moment. With the purpose of test and validate the accuracy achieved by the models, 60% of the samples of each cut have been randomly selected and used as a training set, while the rest are reserved as validation (20%) and test (20%) sets.

Due to the resolution and wide range of numerical representation required in the training of an ANN, this process is performed out of the target processing device (an FPGA). For this task, models have been implemented using a 32-bit floating point numerical representation and trained on an AMD Ryzen 5 3400G CPU at 3.7 GHz computer with a NVIDIA Quadro P2000 GPU. The framework used is the Python-based PyTorch 1.8.1 [25] using the Adam optimizer [26], and training during 50 epochs using a 3·10^−4^ learning rate and a batch size of 1. Additionally, as it is a regression model, the Mean Square Error (MSE) metric is used for cost estimation, whose definition is:

(4) $$\mathrm{MSE}\left(y,\;y^{\prime }\right)=\frac{1}{N}\overset{N-1}{\underset{i=0}{\sum }}{\left(y_{i}-y_{i}^{\prime }\right)}^{2}$$

where y is the actual TVC (whose elements are denoted $y_{i}$), $y^{\prime }$ is the estimated TVC (whose elements are denoted ${y_{i}}^{\prime }$) and N the number of samples.

### 2.4. Low-Cost FPGA System Implementation

Once trained, the neural model must be simplified for its implementation into the selected low-cost portable device. Model simplification includes several considerations, as the selection of a low-complexity non-linear activation function for neural processors, or the numerical resolution providing a tradeoff between size of number representation/results accuracy. For the implementation of the neural model in the FPGA the Vivado High-Level Synthesis (HLS) 2019.2 tool has been used, enabling the definition of the logic system to be implemented from an algorithmic description through a high-level programming language as C++. This tool supports the selection of different datatypes which can be applied to represent the ANN parameters, data inputs and outputs, thus allowing the management of the computational resources of the FPGA while controlling the accuracy loss in the operations of the network. Due to the high complexity required by the implementation of arithmetic operations using floating-point numerical representation compared to the much simpler implementation in fixed-point format, the proposed system is based on a fixed-point representation.

Besides, there is another consideration that allows a further model reduction. In the Section 2.2, when the network architecture was presented, the LReLU was introduced as the solution to the convergence problem known as ‘dying ReLU’. In the physical implementation of the network model, once trained, the LReLU has been replaced by the ReLU, since its implementation is computationally simpler, removing the need of additional multiply operations in the case of negative inputs to the non-linear function.

**Figure 5:**
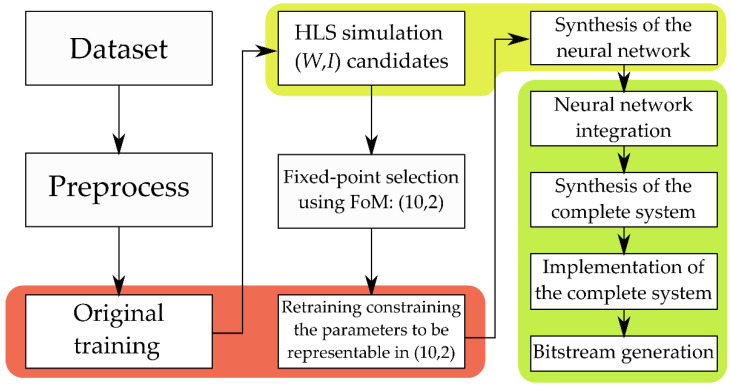
Complete workflow diagram. The steps in the red containers use PyTorch to train the models. The steps where HLS is used to simulate and synthetize the neural network are marked in yellow. Finally, the steps where the complete system (neural network connected with the Processing System) is designed, synthetized, and implemented using Vivado are highlighted in green.

A complete diagram with the implementation steps and the tools used is drawn in Figure 5 and a description of the workload of this process is included in Appendix A.

#### 2.4.1. Fixed-Point Datatype Selection

**Figure 6:**
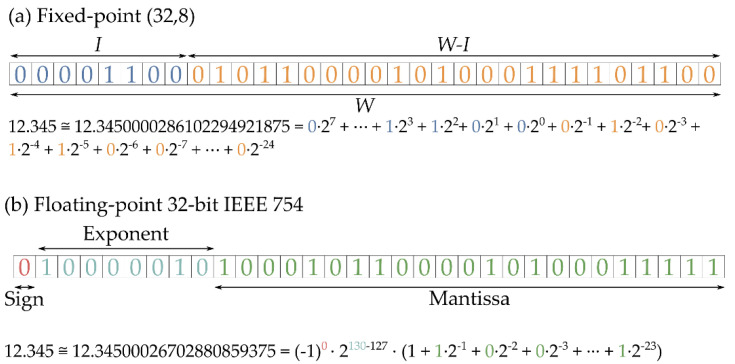
A comparison between the (32,8) fixed-point representation (**a**) and the 32-bit IEEE 754 floating-point representation (**b**).

**Figure 7:**
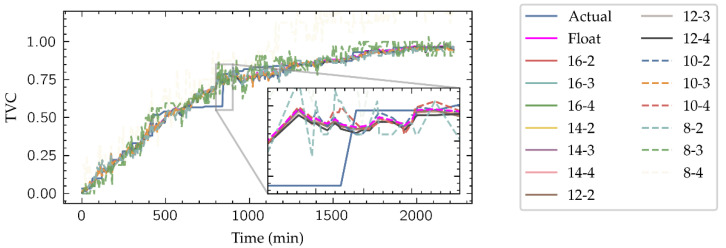
Outputs of the HLS simulation of the neural network for Inside-Outside cut using the different fixed-point candidate datatypes.

As shown in Figure 6, fixed-point datatypes are defined by two parameters: *W* is the total number of bits required to represent a radix-10 number, and *I* is the number of bits representing the integer part, thus denoting a specific fixed-point type as (*W*, *I*). Clearly, *W-I* corresponds to the number of bits dedicated to representing the decimal part of the number. Datatypes with a large number of bits have a direct impact in the FPGA resources consumption, not only on the memory required to their storage but also in the arithmetic structures which size depends on the length of their inputs. To select which fixed-point representation is better for this application, HLS simulations of the implemented network with different (*W*, *I*) pair candidates have been launched, with *W* ranging in the evens from 8 to 16 and *I* ranging from 2 to 4. An example of these simulations is presented in Figure 7, where the estimation for the Inside-Outside cut TVC level given by the model is shown.

**Figure 8:**
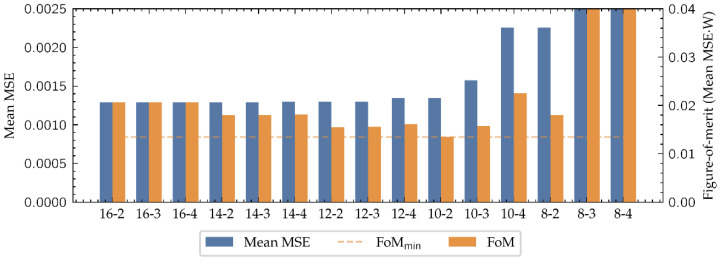
Comparison of the fixed-point candidate datatypes to represent the neural network. The mean error of each datatype is an average of all the MSE computations along the cuts.

To determine a suitable numerical representation for this problem, the MSE between the outputs provided by the system in each (*W*, *I*) candidate representation and the actual TVC curve have been computed, thus obtaining the error associated to each (*W*, *I*) pair. Although datatypes with bigger *W* get a better performance, as shown in Figure 8, they have bigger impact in the FPGA resources consumption, reducing the maximum size of the network that can be implemented and slowing down its operation. To take this into account, a figure-of-merit (FoM) has been defined as:

(5) $$\mathrm{FoM}=\bar{\mathrm{MSE}}\cdot W$$

where $\bar{\mathrm{MSE}}$ is the average MSE along all the cuts and *W* the number of total bits of the candidate representation. This FoM provides a balance between performance and resources consumption, that could be used to choose the datatype which suits the best in any regression problem algorithm. As it is presented in Figure 8, considering the proposed FoM the best option is (*W*, *I*) = (10, 2).

#### 2.4.2. Model Re-Training

For a more adequate model fit, after training in floating-point number representation and applying complexity reduction by means of the fixed-point adaptation of the models, a re-training of 10 epochs has been launched for each of the reduced models using the same hyperparameters as in the original training. In this case, the process adjusts the weights so that they can be represented in the selected data type, (10, 2), thus optimizing the results of the ANN once implemented in the FPGA.

#### 2.4.3. FPGA Selection

The hardware selected in this work to map the developed MLP is a Xilinx Zynq 7020 System on-Chip (SoC) (XC7Z020). This device consists of a Programmable Logic (PL) that includes 85 K programmable logic cells, 53.2 K Look-Up Tables (LUTs), 106.4 K Flip-Flops (FFs), 4.9 Mb of Block Random Access Memory (BRAM) and 220 Digital Signal Processing (DSP) slices of 18 × 25 Multiplier-Accumulator (MACCs) blocks. Additionally, the SoC also includes a Processing System (PS) consisting in a dual-core ARM Cortex-A9 with a maximum 667 MHz clock frequency and 512 MB RAM.

**Figure 9:**
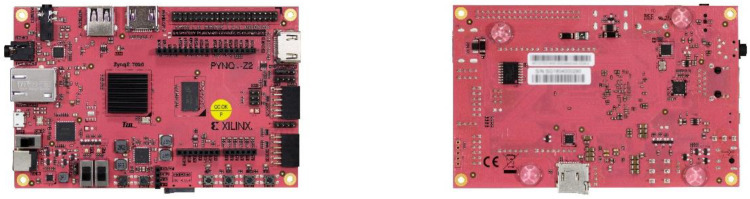
PYNQ-Z2 board mounting the Zynq 7020 SoC.

The development board used is a PYNQ-Z2 from TUL (Figure 9), specifically designed to support the Python Productivity for Zynq (PYNQ) framework, an open-source project from Xilinx based on Python that eases the use of Zynq-based platforms. In this project the PYNQ 2.4 image has been used, based on Ubuntu 18.04.

#### 2.4.4. ANN Synthesis

Once the suitable fixed-point datatype has been selected and the parameters of the models have been readjusted to comply the constraint of being representable in that format, the ANN is ready to be mapped from a high-level C++ algorithmic description to its physical implementation, by appropriately connecting the logic resources in the targeted FPGA, a Xilinx XC7Z020.

The first step is to synthesize the ANN using the HLS tool, that allows including directives in this process that enable the control of how the high-level algorithm is mapped into the device hardware. Two of these directives are *unroll* and *pipeline*. Unroll directive allows the implementation of loops in the algorithm to run in parallel in the target hardware, thus reducing the total time in their execution at the cost of a higher use of resources. On the other hand, pipeline directive tries to force HLS to complete each loop iteration in a given number of clock cycles (usually one). Since the same hardware is being reused in each loop cycle, this directive reduces the time to complete the loop at a low increase of resources consumption. For the ANN design of this work, whose layers have the algorithmic description shown in Code 1, an *unroll* in the inner loops of each layer and a *pipeline* in the external ones have been found to be the best option, highly reducing the latency of the system, i.e., the inference time.

**Code** **1.** *Example of the high-level algorithmic description of a fully-connected layer operation (C++ implementation of Equation (2)).*

ExtLoop: for(i = 0; i < OutDim; i++)

{

        tmp = b[i];

        IntLoop: for(j = 0; j < InDim; j++)

        {

                tmp + = W[j][i]*x[j];

        }

        y[i] = ReLU(tmp);

}

#### 2.4.5. Synthesis, Implementation, and Exportation of the Complete System

**Figure 10:**
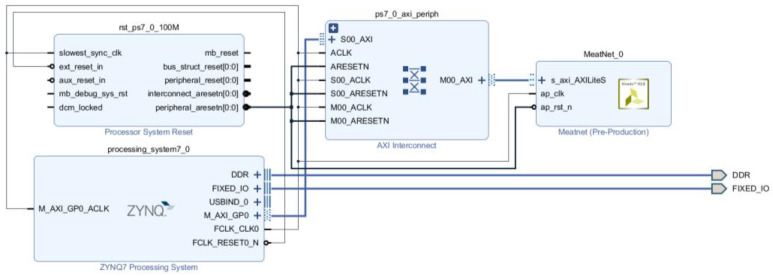
Block design with the ANN block integrated in the Zynq PS.

Once the final synthesis directives have been applied and this process is completed, the system is ready to be exported to a FPGA. For this, the Vivado HLS tool generates an IP (Intellectual Property) module in a specific Hardware Description Language (HDL) which models the synthetized system in terms of electronic signals that flow between the different electronic components (registers, arithmetic/logic circuits). This description, close to the electronic architecture of the final system, can be easily integrated by the Vivado main tool as a component in the block design of the full architecture, that must include some additional elements as the Zynq Processing System IP block (Figure 10). This module describes the electronic interface required to provide the suitable channels from outside to the FPGA, for input the signals that will be processed by the implemented ANN and output its corresponding responses. The autoconnection feature of Vivado eases the connection of the PS and ANN modules, creating another two auxiliary blocks: the reset module and the peripherals interconnection module.

Then, the complete system can be synthesized in a process that transforms the IP-based design into a low-level description based on logic gates. The implementation process then maps this gate-level description to the resources of the selected FPGA, optimizing the location of the electronics in the available CLBs and their interconnection (routing) so that the time delay between the input to the system of a stimuli vector and the output of the corresponding response is minimized. Finally, after the global synthesis and the implementation processes, the bitstream file that contains the information of the configuration can be generated to be downloaded in the FPGA, thus building the tailored hardware design.

### 2.5. Hardware Interface

Once the designed ANN is physically implemented into the FPGA, a performance test has been made to provide information about its features. For this, a Jupyter notebook running in the CPU of the Zynq SoC interfaces to the neural network in the FPGA. Firstly, the bitstream describing the ANN mapping is uploaded into the PYNQ-Z2 file system and then it is loaded in the FPGA using the *overlay* module of the *pynq* Python package [27]. This module includes the drivers needed to control the designed IP, in this case the ANN, using Memory-Mapped Input/Output (MMIO) to write and read in the IP ports from the CPU available in the SoC. As the port drivers included in the *overlay* module do not support fixed-point data transmission, a custom driver has been developed, that manually writes the inputs and network parameters an reads the outputs, making the interaction with the FPGA-implemented neural network from the Jupyter environment straight-forward: input the stimuli data, read the estimated TVC, etc.

## 3. Results

### 3.1. ANN Synthesis Results

Applying the synthesis directives commented in the Section 2.4.4, the inference process for a single input pattern requires 158 clock cycles. The minimum clock period for this implementation is 11.06 ns, thus resulting in an inference time of 1.747 µs. Additionally, Table 2 presents the FPGA resources occupation, showing how the developed system fits smoothly in the PL of the Zynq 7020.

### 3.2. Regression Task

**Figure 11:**
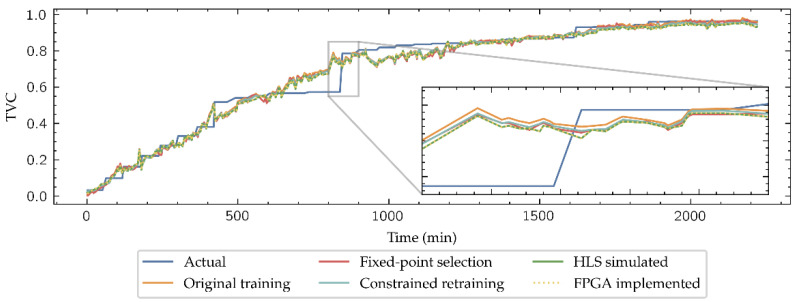
Inside-Outside regression outputs of all the model steps.

Figure 11 shows the results of testing the model developed using the Inside-Outside data subset at the different implementation stages: the original-trained model, the HLS simulation output of the (10, 2) format during the fixed-point selection, the results of the retrained model with constraints inferring from PyTorch, its results in the HLS simulation, and its implementation in the FPGA. As the neural network is designed to estimate the TVC as a regression model, the MSE of each model in its five stages for the 12 beef cuts and its average value ($\bar{\mathrm{MSE}}$) is presented in Table 3.

As expected, the MSE increases in general in the HLS simulated models, which includes fixed-point datatypes, compared to the high-level simulated models in PyTorch with floating-point number representation. It is also remarkable that the HLS simulations provide identical TVC estimation than physical implementation of the models, which allows to establish the results expected in the FPGA implementation even prior to ending the implementation workflow, saving development time. Finally, although the retraining does not improve the implemented models compared to the HLS simulation during the fixed-point selection by a slight difference, results are improved in the classification task, so that re-training is justified in the model development.

### 3.3. Classification Task

As commented in Section 2, the dataset includes a four-category quality label, depending on the *TVC* range, using the following expression:

(6) $$\mathrm{Label}\left(TVC\right)=\{\begin{array}{c} \begin{array}{c} 1\;\mathrm{if}\;TVC<3\;\;\;\;\\ 2\;\mathrm{if}\;3\leq TVC<4 \end{array}\\ \begin{array}{c} 3\;\mathrm{if}\;4\leq TVC<5\\ 4\;\mathrm{if}\;5\leq TVC\;\;\;\; \end{array} \end{array}$$

This makes the network can work not only as a regression model but also as a classifier. Table 4 shows the classification metrics obtained in each model development stage for all the cut samples and their average. Accuracy is defined as:

(7) $$\mathrm{Accuracy}\left(y,\;y^{\prime }\right)=\frac{1}{N}\overset{N-1}{\underset{i=0}{\sum }}\delta \left(y_{i},\;y_{i}^{\prime }\right)$$

where y is the true labels vector (whose elements are denoted as $y_{i}$), $y^{\prime }$ the predicted labels vector (whose elements are denoted as ${y_{i}}^{\prime }$), N the number of samples and $\delta \left(y_{i},\;y_{i}^{\prime }\right)$ is the Kronecker delta that is valued 1 if $y_{i}=y_{i}^{\prime }$ and 0 if not. The maximum value of the accuracy is 1, when the classifier has perfectly matched all the samples; and value 0 indicates it has not accurately predicted any.

The other metric shown in Table 3 is the Macro F1 score, given by:

(8) $$\mathrm{Macro}\;F1\left(y,\;y^{\prime }\right)=\frac{1}{L}\overset{L-1}{\underset{l=0}{\sum }}F1\left(y_{l},\;y_{l}^{\prime }\right)$$

where L is the number of labels, $y_{l}$ the subset of true labels belonging to the l label, ${y_{l}}^{\prime }$ the subset of predicted labels belonging to the l label, and $F1\left(y_{l},\;y_{l}^{\prime }\right)$ is the F1 score, whose expression is:

(9) $$F1\left(y_{l},\;{y_{l}}^{\prime }\right)=2\frac{{\mathrm{Precision}}_{l}\cdot {\mathrm{Recall}}_{l}}{{\mathrm{Precision}}_{l}+{\mathrm{Recall}}_{l}}\;$$

where precision is defined as:

(10) $${\mathrm{Precision}}_{l}=\frac{{\mathrm{True}\;\mathrm{Positives}}_{l}}{{\mathrm{True}\;\mathrm{Positives}}_{l}+{\mathrm{False}\;\mathrm{Positives}}_{l}}$$

and recall:

(11) $${\mathrm{Recall}}_{l}=\frac{{\mathrm{True}\;\mathrm{Positives}}_{l}}{{\mathrm{True}\;\mathrm{Positives}}_{l}+{\mathrm{False}\;\mathrm{Negatives}}_{l}}$$

The macro F1 score can be interpreted as the average along all the labels of the weighted harmonic mean of the precision and the recall at each label, reaching the best value at 1 and its worst at 0.

**Figure 12:**
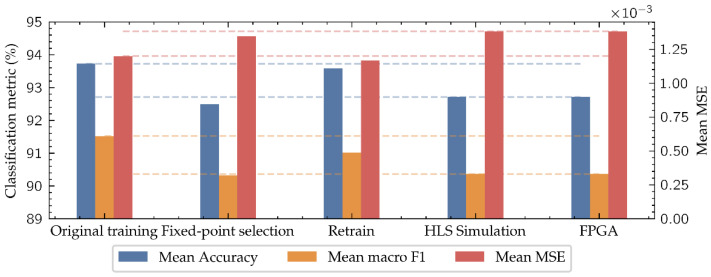
Evolution of the mean metrics of both tasks along all the model implementation stages. The dashed lines mark the difference between the original model and the implemented one.

As shown in Figure 12 and Table 4, the model mean accuracy after the original training with PyTorch is 93.73% and the mean macro F1 score is 91.52%, which are the values that a computer would achieve for this architecture following the same training strategy. When the parameters of these models are discretized using the (10, 2) datatype during the fixed-point selection, for its application in the HLS simulation, performance decreases reducing the mean accuracy to 92.49% and the mean macro F1 score to 90.33%. This is in part due to the loss of accuracy in the (10, 2) number representation format compared to the floating-point values of the original training parameters. To address this issue, a retraining process is carried out, improving the model accuracy of the final implementation, achieving a 92.72% in mean accuracy and 90.36% in mean macro F1 score. In this way, the difference in performance between the final implemented model on the low-cost FPGA and the computer-based model is reduced to 1.01% in mean accuracy and 1.16% in mean macro F1 score, negligible enough to consider the implementation of this type of algorithms on low-cost FPGAs when in-situ applications are demanded.

As Table 4 and Figure 12 show, the network retraining improves the performance in the classification task, while it becomes worse in the regression task. This is because the retraining improves the network performance, especially near the classes’ boundaries where the classification errors are concentrated, keeping this improvement to the end model.

## 4. Conclusions

A low-cost FPGA-based Machine Learning tool has been developed for in-situ food quality determination using e-nose VOCs detection to estimate the TVC. It is based on a fully connected neural network that is implemented in edge-computing-oriented devices, enabling through its application real-time quality tracking along all the food production and distribution chain.

Compared to the traditional TVC determination methods, as PCR, FTIR or hyperspectral imaging, its estimation through VOCs detection using a e-nose represents a low-cost alternative that could be widely used in the industry. Besides, its implementation in an embedded device enables the in-situ operation everywhere, removing the requirement of internet connection that is necessary for cloud computing solutions. For this reason, special attention has been paid in the implementation process, customizing the original model to reduce its impact in the FPGA resources consumption, carefully selecting the fixed-point datatype to represent the network and using a simpler activation function, thus enabling its mapping into the resources of a low-cost and low-size FPGA. Besides, this thorough implementation process combined with the selection of a small and relatively simple neural architecture makes the edge-device to get results close to its analogous implementation in bulk computers.

In this case, the model was trained to estimate the TVC of a determined beef cut using the readings of an e-nose, using the data available in [17], but any other biosensor fusion could be addressed using the same workflow. Firstly, the models were trained using a high-level neural network framework, presenting a mean accuracy of 93.73%. After this, the implementation process was started. As the targeted low-cost FPGA, the Xilinx Zynq 7020 (XC7Z020), reduced number of resources, the impact in the resources consumption of the neural architecture must be controlled. To enable this, the representation of the neural network with fixed-point datatypes has been considered. After a fixed-point selection process using an adequate figure-of-merit (Equation (5)), the (10, 2) datatype, with 10 bits, in total of which two were dedicated to the integer part, is chosen, which made the models score a 92.49% in mean accuracy, slightly reducing the performance with respect to the original trained models. In order to reduce this performance loss, due to the impact of the difference of the floating-point trained weights with their nearest representation using (10, 2), a retraining process was launched, in which they were constrained to be representable in the selected datatype. Using the retrained parameters, the (10, 2)-implemented models presented 92.72% in mean accuracy, slightly improving the early-implemented model during the datatype selection process. Finally, the implementation process was completed, achieving all the necessary steps to generate the bitstream file, which builds the neural architecture in the FPGA, where the model performance was validated using a custom driver finding the same performance as in the HLS simulation.

It is clear that the results may be improved by targeting a bigger FPGA, as one of the Zynq Ultrascale+ series, which would allow to implement larger and more complex neural architectures or more robust Machine Learning algorithms, while maintaining the portability and low-cost objectives.

## Figures and Tables

**Table 1:** Main sensitivities of the e-nose sensors.

Sensor	Main Sensitivity	Sensor	Main Sensitivity
MQ135	NH_3_, NO_x_, CO_2_, alcohol, benzene, smoke	MQ3	Alcohol
MQ136	H_2_S	MQ4	Methane, propane, butane
MQ137	NH_3_	MQ5	H_2_, LPG, methane, CO, alcohol
MQ138	n-hexane, benzene, NH_3_, alcohol, smoke, CO	MQ6	Methane, butane, propane, LPG and natural gas
MQ2	Liquefied Petroleum Gas (LPG), i-butane, propane, methane, alcohol, H_2_, smoke	MQ8	H_2_
		MQ9	Methane, carbon monoxide and propane, LPG

**Table 2:** FPGA resources consumption.

Resource	Block RAM	Digital Signal Processing	Flip-Flops	Look-Up Tables
Total	98	55	7256	9771
Available	280	220	106,400	53,200
Utilization (%)	35	25	6	18

**Table 3:** Mean Square Error in each stage of the models. All the values have a scale of 10^−3^.

Model	Cut												Mean
	Inside Outside	Round	Top Sirloin	Tenderloin	Flap Meat	Striploin	Rib Eye	Skirt Meat	Brisket	Clod Chuck	Shin	Fat	
Original training ^1^	2.25	0.75	1.36	1.18	1.04	2.22	0.43	0.42	0.95	2.01	0.91	0.88	1.20
Fixed-point selection ^2^	2.33	0.96	1.41	1.18	1.45	2.51	0.52	0.53	1.09	2.09	1.06	1.01	1.35
Re-training ^1^	2.07	0.85	1.42	1.16	0.96	1.93	0.43	0.44	1.05	2.03	0.78	0.88	1.17
HLS Simulation ^2^	2.09	1.61	1.45	1.34	1.29	2.14	0.49	0.48	1.43	2.31	0.98	0.97	1.38
FPGA	2.09	1.61	1.45	1.34	1.29	2.14	0.49	0.48	1.43	2.31	0.98	0.97	1.38

^1^ Results obtained with PyTorch evaluation using floating-point datatypes. ^2^ Results obtained with an HLS simulation using fixed-point datatypes.

**Table 4:** Classification metrics in each stage of the models. All the values are expressed in percentage (%).

Model	Metric	Cut												Mean
		Inside	Round	Top Sirloin	Tenderloin	Flap Meat	Striploin	Rib Eye	Skirt Meat	Brisket	Clod Chuck	Shin	Fat	
		Outside												
Original	Accuracy	87.16	95.95	94.59	96.17	95.27	92.57	96.4	95.95	94.37	91.22	94.37	90.77	93.73
training ^1^	Macro F1	87.1	94.65	92.11	94.72	93.12	89.83	93.75	92.87	91.77	89.47	91.9	86.99	91.52
Fixed-point	Accuracy	84.68	93.24	94.14	97.07	90.54	91.89	93.47	95.5	94.37	90.32	94.14	90.54	92.49
selection ^2^	Macro F1	85.27	90.32	91.56	96.17	88.15	91.21	88.67	92.51	92.01	89.43	91.67	86.95	90.33
Retrain ^1^	Accuracy	87.61	94.82	92.12	96.17	94.82	93.69	93.47	96.4	94.59	89.41	95.5	94.37	93.58
	Macro F1	87.92	92.71	87.47	94.44	92.04	91.31	88.51	93.79	92.46	86.83	93.01	91.76	91.02
HLS Simulation ^2^	Accuracy	85.14	94.37	92.34	94.82	93.92	92.34	93.92	96.85	93.47	89.19	93.02	93.24	92.72
	Macro F1	85.25	92.43	88.48	92.44	91.47	91.01	89.29	94.45	91.18	87.03	90.01	91.31	90.36
FPGA	Accuracy	85.14	94.37	92.34	94.82	93.92	92.34	93.92	96.85	93.47	89.19	93.02	93.24	92.72
	Macro F1	85.25	92.43	88.48	92.44	91.47	91.01	89.29	94.45	91.18	87.03	90.01	91.31	90.36

^1^ Results obtained with PyTorch evaluation using floating-point datatypes. ^2^ Results obtained with an HLS simulation using fixed-point datatypes.

## Data Availability

The data used in this study are openly available in Dataset for Electronic Nose from various beef cuts at 10.21227/596e-xn25, reference number [17]. There is a release of the open-source code used in this work, available in https://github.com/eneriz-daniel/MeatNet accessed on 31 August 2021.

## Appendix A

The authors would like to include some lines to give the reader an idea of how much work is needed to develop the system presented in this work. The first training and the retraining with the constrained parameters are completed with PyTorch, which eases the implementation of the neural networks in few lines of code.

The hardest part of the project is the C++ implementation of the neural architecture, which must render the PyTorch version. This task has been achieved manually using layers as the described in Code 1. Since it is the input for the HLS tool, enabling its synthesis and future implementation, the usage of libraries is not well-supported, thus a pure C/C++ implementation is mandatory. Additionally, a testbench program must be also developed to run the HLS simulations, where the evaluation of the dataset is included, thus evaluating its performance when implemented. Once the C/C++ version of the network and its testbench are developed, the HLS simulations can be launched automatically from Python parsing the C/C++ codes and using Tool Command Language (Tcl) scripts.

After the simulation, the next step in the HLS workflow is the synthesis, where the Vivado HLS Graphic User’s Interface (GUI) is especially helpful since it creates a time chart of the operations sequence after each synthesis, thus enabling the visualization of points of improvement that can be addressed with synthesis directives. This must be conducted manually and carefully. A good design synthesized using HLS is marked to have a good time analysis that had motivated the synthesis directives.

Once the HLS system is synthesized, the IP export can be launched, which is done automatically. Then its integration in the Vivado main tool is relatively simple since the automatic interconnection feature of this program eases the work to get a block design as the displayed in Figure 10. After this, the complete synthesis, implementation and bitstream generation are launched, which do not require any supervision.

Finally, there is the driver to interface the FPGA implemented system and the SoC CPU. As commented in Section 2.5, although the *pynq* package should include drivers that automatically detect the ports in an integrated design, they do not support the usage of fixed-point datatypes. To address this problem, we have developed a custom driver that manages this kind of representation, allowing to easily load of parameters and infer results.

As a summary and with the intention of illustrate better the balance between the automatic and manual task, the authors estimate that 60% of the time invested in the development of this project was spent in manual tasks, as coding the implementations of the network or developing the interface driver, while the remaining 40% was dedicated to the supervision of the automatic tasks, as the HLS simulation and synthesis or the implementation process of the complete system in the Vivado main tool.
